# Mitochondrial dysfunction and lipid droplet dynamics in MASH: HSD17B13 as a therapeutic target

**DOI:** 10.3389/fcell.2026.1912237

**Published:** 2026-07-22

**Authors:** Shanzab Noor, Yuan Tian, Wen Su

**Affiliations:** 1 Department of Pathophysiology and Pathology, Shenzhen University Medical School, Shenzhen, China; 2 Department of Biochemistry and Molecular Biology, Shenzhen University Medical School, Shenzhen, China

**Keywords:** HSD17B13, lipid droplet–mitochondria axis, MASH, metabolic dysfunction-associated steatohepatitis, MASLD, resmetirom, RNA interference, semaglutide

## Abstract

Metabolic dysfunction-associated steatohepatitis (MASH) is increasingly recognized as a disorder of inter-organelle communication, in which the lipid droplet (LD)–mitochondria interface serves as a central metabolic hub. Under physiological conditions, this interface couples LD lipolysis to mitochondrial β-oxidation, ensuring that fatty-acid release matches energy demand. In MASH, chronic nutrient excess disrupts this coupling, driving the accumulation of lipotoxic metabolites, activating innate immune pathways, and perpetuating hepatocellular injury and inflammation. Among the proteins proposed to operate at this LD-mitochondria interface, hydroxysteroid 17β-dehydrogenase 13 (HSD17B13) has emerged as a particularly compelling candidate. A loss-of-function human genetic variant is associated with reduced risk of chronic liver disease, motivating therapeutic development; however, whether HSD17B13 directly governs physical organelle apposition or merely influences lipid flux remains unresolved, highlighting a key gap between human genetic evidence and experimental models. This review synthesizes current understanding of the molecular organization of the LD-mitochondria axis, critically examines the proposed scaffolding and enzymatic functions of HSD17B13, and discusses the therapeutic potential of restoring organelle communication as a unified strategy in MASH. We conclude that targeting inter-organelle interfaces, rather than isolated metabolic reactions, offers a genetically supported and mechanistically rational path forward.

## 1 Introduction

Metabolic dysfunction-associated steatotic liver disease (MASLD), the current nomenclature for the condition previously termed non-alcoholic fatty liver disease, affects an estimated 38% of adults worldwide and is projected to exceed 55% by 2040 (Powell et al., 2021). Within this spectrum, metabolic dysfunction-associated steatohepatitis (MASH) is histologically defined by steatosis, hepatocyte ballooning and lobular inflammation, with or without concomitant fibrosis. MASH has become an increasingly common indication for liver transplantation in the United States and a major contributor to MASLD-associated hepatocellular carcinoma (HCC) (Lonardo et al., 2019; Collaborators, 2026). Decision-analytic modelling predicts that the prevalence of MASH in United State adults will increase from 5.8% in 2020 to 7.9% in 2050, accompanied by rising cirrhosis-related mortality and HCC incidence (Collaborators, 2026). The occurrence of MASH in non-obese individuals indicates that adiposity alone is insufficient to account for disease progression, underscoring the urgent need to delineate the underlying molecular mechanisms (Eslam and George, 2020).

Conceptual models of MASH pathogenesis have accordingly evolved beyond the original “two-hit” hypothesis towards a “multiple-parallel-hits” framework, in which insulin resistance, enhanced hepatic *de novo* lipogenesis (DNL), augmented adipose-derived non-esterified fatty-acid (NEFA) flux, and impaired fatty-acid oxidation (FAO) operate concurrently (Ti et al., 2021). Under physiological conditions, hepatocytes balance lipid input-derived approximately 60% from adipose-tissue lipolysis, 25% from DNL, and 15% from dietary sources, against three principal fates: storage as triacylglycerol (TAG) in LDs, secretion as very-low-density lipoprotein (VLDL), and mitochondrial β-oxidation (Hodson and Gunn, 2019; Hansen et al., 2025). In MASH, SREBP-1c- and ChREBP-driven DNL is increased, adipose insulin resistance augments NEFA delivery, and mitochondrial oxidative capacity becomes insufficient to handle the combined substrate burden (Pinkosky et al., 2020; Iturbe-Rey et al., 2025). The consequent accumulation of ceramides, diacylglycerols and lysophosphatidylcholines activates MAPK and NF-κB signalling, promotes Kupffer-cell activation and drives hepatic stellate-cell (HSC) transdifferentiation, thereby linking steatosis to inflammation and fibrosis (Friedman et al., 2018).

A comprehensive understanding of these pathological processes necessitates close consideration of the physical and metabolic interface between LDs and mitochondria (Benador et al., 2018; Benador et al., 2019). LDs are dynamic organelles bounded by a phospholipid monolayer and decorated by a specialized proteome that includes perilipins, CIDE-family proteins and several short-chain dehydrogenases (Bersuker and Olzmann, 2017; Henne and Cohen, 2026). Mitochondria are physically and metabolically coupled to LDs through complexes such as PLIN5–MFN2, VPS13D–TSG101 and ACSL1–SNAP23, which facilitate the channelling of lipolysis-derived fatty acids into mitochondrial β-oxidation pathways (Wang et al., 2021; Freyre et al., 2019; Young et al., 2018). These contacts integrate cellular energy status via AMPK, hormonal signals through β-adrenergic and PKA pathways, and stress responses through mitophagy and endoplasmic-reticulum (ER) crosstalk. Accumulating evidence thus places disruption of the LD-mitochondria interface among the early mechanistic abnormalities contributing to MASH progression (Sassano et al., 2017; Goodpaster and Sparks, 2017).

Within this framework, HSD17B13 has emerged as one of the most robustly genetically supported targets for pharmacological intervention. HSD17B13 is a hepatocyte-enriched, LD-monolayer-associated member of the short-chain dehydrogenase/reductase (SDR) superfamily and has been implicated in both LD expansion, through effects on the SREBP-1c axis, and LD turnover, through PKA -dependent regulation of ATGL–CGI-58-mediated lipolysis (Su et al., 2014; Su et al., 2019; Su et al., 2022). An exome-wide association study by Abul-Husn and colleagues identified the splice variant rs72613567: TA, which yields a truncated and unstable protein, as protective against chronic liver disease (Abul-Husn et al., 2018; Pirola et al., 2019). In combination with PNPLA3 p. I148M and TM6SF2 p. E167K, HSD17B13 genotype also contributes to polygenic risk estimates for cirrhosis and HCC (Gellert-Kristensen et al., 2020). This human genetic evidence has prompted clinical development of GalNAc-siRNA agents, including GSK4532990/ARO-HSD and ALN-HSD, and selective small-molecule inhibitors such as BI-3231 and INI-822 (Mak et al., 2023; Sanyal et al., 2025; Thamm et al., 2023).

This review has three principal objectives. First, we summarize the molecular architecture of the hepatocyte LD-mitochondria axis, with emphasis on tethering complexes, mitochondrial dynamics, organelle quality control, and metabolic integration. Second, we evaluate HSD17B13 as a regulatory node within this axis, addressing its structure, proposed lipogenic and lipolytic functions, multilayered regulation, and the divergence between human genetic data and rodent phenotypic observations. Third, we discuss the emerging therapeutic landscape and the rationale for combining HSD17B13-directed interventions with thyroid hormone receptor-β activation, GLP-1 receptor agonism or FXR/PPAR modulation. Particular attention is given to unresolved issues, including the physiological substrate of HSD17B13, the relative contributions of its scaffolding and catalytic activities, and the current lack of biomarkers that directly report LD-mitochondria contact-site integrity.

## 2 Mitochondrial dynamics, quality control and inflammatory signalling

Hepatocytes contain approximately 1,000–2,000 mitochondria (An et al., 2020), which collectively generate the ATP required for gluconeogenesis, ureagenesis, lipoprotein assembly, and bile secretion (Wen et al., 2025). Hepatic mitochondrial homeostasis is maintained by coordinated control of organelle morphology, selective clearance of damaged mitochondria, and transcriptional regulation of nuclear-encoded mitochondrial proteins. In MASH, disruption of each of these processes amplifies the others, producing a combined bioenergetic and signalling defect that contributes to disease progression.

### 2.1 Mitochondrial dynamics in hepatocytes

Mitochondrial morphology is governed by opposing GTPase-dependent fission and fusion machineries (Ishihara et al., 2013; Chan, 2020). Fission is mediated by cytosolic dynamin-related protein 1 (DRP1), which is recruited to outer-mitochondrial-membrane receptors via four receptors including mitochondrial fission factor (MFF), fission protein 1 (FIS1), and mitochondrial dynamics proteins of 49 kDa and 51 kDa (MID49 and MID51). At these sites, DRP1 oligomerizes into helical assemblies that constrict the organelle in a GTP hydrolysis-dependent manner (Jin et al., 2021). Fusion is orchestrated by the outer-membrane mitofusins MFN1 and MFN2 for outer-membrane fusion and the inner-membrane GTPase OPA1 for matrix mixing and cristae remodeling. In hepatocytes, MFN2 additionally cooperates with PLIN5 to tether LDs to mitochondria, coupling lipolysis to mitochondrial β-oxidation (Wang et al., 2011; Joaquim et al., 2025).

**FIGURE 1:**
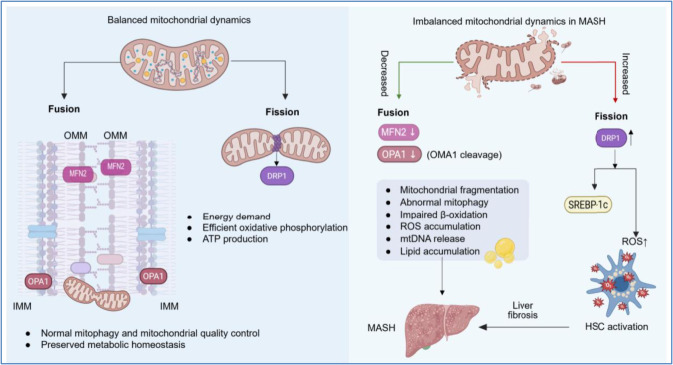
Mitochondrial dynamics in healthy hepatocytes and MASH. In healthy hepatocytes, balanced mitochondrial fusion and fission maintain mitochondrial network integrity, and efficient oxidative phosphorylation, supported by normal mitophagy. In MASH, DRP1 hyperactivation, MFN2 downregulation, and OMA1-dependent OPA1 processing drive excessive mitochondrial fragmentation. This impairs β-oxidation, promotes ROS accumulation, mtDNA release and LD accumulation. mtDNA triggers inflammatory signalling, activating HSCs and promoting liver fibrosis. Impaired mitophagy exacerbates mitochondrial damage, establishing a pathogenic cycle that drives MASH progression.

This dynamic equilibrium is disrupted in MASH. Chronic nutrient excess and pro-inflammatory signaling promote DRP1 activation and mitochondrial fragmentation, whilst simultaneously driving SREBP-1c-dependent lipogenesis and suppressing CPT1a-mediated fatty-acid oxidation (Jin et al., 2021). Concurrently, acetylation-dependent destabilization of MFN2 impairs LD-mitochondria contacts and restricts substrate delivery to oxidative pathways (Wang et al., 2011; Joaquim et al., 2025). OPA1 processing may also be altered in a tissue-dependent manner: oxidative stress activates the inner-membrane protease OMA1, leading to increased short OPA1 isoforms and disrupted cristae architecture in hepatocytes, whereas peripheral blood mononuclear cells from patients with MASH have been reported to exhibit elevated OPA1 expression (MacVicar and Langer, 2016; Gancheva et al., 2022). Collectively, mitochondrial fragmentation, impaired LD coupling and diminished oxidative capacity represent recurrent hallmarks of human MASH and experimental disease models (Mancina et al., 2016; Bhatti et al., 2017) (Figure 1).

### 2.2 Mitochondrial quality control: PINK1–Parkin mitophagy, mitochondrial-derived vesicles and mitocytosis

Mitophagy, the selective autophagic clearance of dysfunctional mitochondria, restrains the accumulation of reactive oxygen species (ROS) and limits the release of mitochondrial damage-associated molecular patterns (DAMPs). In the canonical pathway, PINK1 accumulates on the outer membrane of depolarized mitochondria, recruits Parkin, and promotes ubiquitination of outer-membrane proteins, thereby marking damaged organelles for autophagosomal sequestration. This pathway undergoes adaptive induction during early MASLD but becomes attenuated under sustained dietary stress, thereby permitting the persistence of dysfunctional mitochondria (Han et al., 2023). Parkin-independent mechanisms, including p62-dependent KEAP1/Rbx1 ubiquitination and SIRT3–BNIP3 signaling, provide partial functional redundancy (Yamada et al., 2018), whereas IRGM1 couples mitophagy to lipophagy, and its deficiency sensitizes to MASH (Kaufman and Mora, 2021).

Hepatocytes also employ alternative quality-control routes that operate independently of canonical mitophagy. Mitochondrial-derived vesicles (MDVs), originating from both the outer and inner membranes, deliver oxidized cargo to lysosomes or peroxisomes for degradation. In parallel, severely damaged mitochondria may be expelled from cells through mitocytosis or autophagic secretion, providing an additional layer of defense against organelle dysfunction (Karbowski and Youle, 2011; Prashar et al., 2024; Popov, 2023). Failure of these complementary quality-control mechanisms contributes to the accumulation of ROS-producing mitochondria in advanced MASH, and restoration of these pathways warrants investigation as a potential therapeutic avenue.

### 2.3 The AMPK–SIRT1–PGC-1α energy-sensing axis controls mitochondrial dynamics and quality

The mitochondrial dynamics and quality-control mechanisms are under the control of upstream energy-sensing pathways according to cellular nutrient and energy status. The AMPK–SIRT1–PGC-1α axis co-ordinates these processes. Imbalances between ATP demand and supply are sensed by AMP-activated protein kinase (AMPK), which is allosterically activated by increased AMP/ATP and ADP/ATP ratios and phosphorylated at Thr172 by upstream kinases LKB1 or CaMKK2 (Garcia and Shaw, 2017). Once activated, AMPK suppresses anabolic lipid synthesis through phosphorylation of acetyl-CoA carboxylase (ACC) and inhibition of SREBP-1c, while concurrently promoting fatty-acid oxidation by relieving malonyl-CoA-mediated inhibition of CPT1 (Pinkosky et al., 2020). The AMPK-SIRT1-PGC-1α axis mediates the transcriptional component of this adaptive response. AMPK elevates cellular NAD^+^ availability, thereby activating SIRT1, which deacetylates and activates PGC-1α (Canto et al., 2009). PGC-1α, in turn, coactivates PPARα and other nuclear receptors to induce the expression of FAO enzymes and mitochondrial biogenesis programmes (Halling and Pilegaard, 2020). Hepatocyte-specific deletion of PGC-1α exacerbates steatohepatitis, whereas preservation of this pathway supports antioxidant defenses, redox balance, and antifibrotic signalling (Schlaepfer and Joshi, 2020).

This energy-sensing network is closely integrated with LD metabolism. AMPK modulates perilipin phosphorylation and LD-mitochondria association, and intersects with PKA-dependent phosphorylation of HSD17B13 at Ser33 to regulate TAG hydrolysis (Goodpaster and Sparks, 2017). Thus, chronic nutrient excess subverts AMPK-SIRT1-PGC1α driven metabolic adaptation, impairing mitochondrial dynamics and quality control. This failure sets the stage for the mtDNA-driven inflammatory amplification discussed in Section 2.4.

### 2.4 Mitochondrial DNA stress and inflammatory amplification through cGAS-STING

**FIGURE 2:**
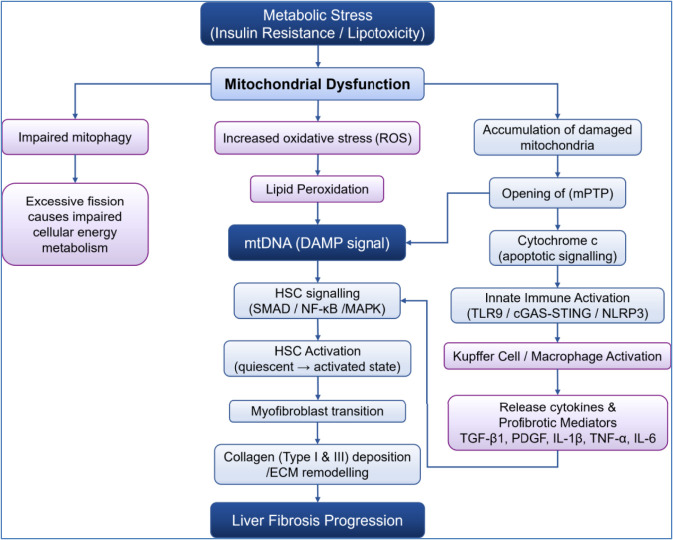
Mitochondria dysfunction as a central driver of inflammatory and fibrotic signaling in MASH. Metabolic stress, induced by insulin resistance and lipotoxicity, initiates a cascade of mitochondrial dysfunction in hepatocytes. This pathogenesis proceeds through three interconnected pathways: (1) impaired mitophagy and excessive fission, leading to compromised cellular energy metabolism; (2) increased reactive oxygen species (ROS) production, resulting in lipid peroxidation; and (3) mitochondrial permeability transition pore (mPTP) opening, which triggers the release of mtDNA (acting as a DAMP signal) and cytochrome c. The subsequent activation of innate immune pathways including TLR9, cGAS–STING, and the NLRP3 inflammasome, drives Kupffer cell and macrophage activation. This inflammatory milieu promotes the secretion of cytokines and profibrotic mediators (e.g., TGF-β1, PDGF, IL-1β), which orchestrate hepatic stellate cell (HSC) activation, myofibroblast transition, and extracellular matrix (ECM) remodeling, ultimately culminating in liver fibrosis progression.

Mitochondrial DNA (mtDNA) serves both as a target of metabolic stress and as a mediator of inflammatory signaling in MASH (Figure 2). An increased mtDNA mutational burden, particularly within oxidative-phosphorylation genes, has been associated with greater histological severity, and selected cytochrome-b variants have been linked to advanced fibrosis (Begriche et al., 2013). Epigenetic alterations, including mtDNA hypermethylation and repression of ND6 transcription, may further compromise complex I activity (Mposhi et al., 2023). Calcium overload and ROS can trigger mitochondrial permeability transition and mtDNA release into the cytosol, where it activates cGAS–STING and TLR9 signalling; concomitantly, mitochondrial ROS prime the NLRP3 inflammasome. The ensuing production of TNF-α, IL-1β and IL-6 by Kupffer cells and recruited macrophages drives HSC activation via SMAD, NF-κB and MAPK pathways, establishing a feed-forward loop that links metabolic stress to progressive fibrosis (Friedman et al., 2018).

Critically, this inflammatory cascade originates upstream from defects in mitochondrial dynamics and quality control. Accordingly, therapeutic strategies that restore fusion–fission balance or mitophagy flux may complement LD-directed approaches.

## 3 Lipid droplet biogenesis, dynamics and proteome

**FIGURE 3:**
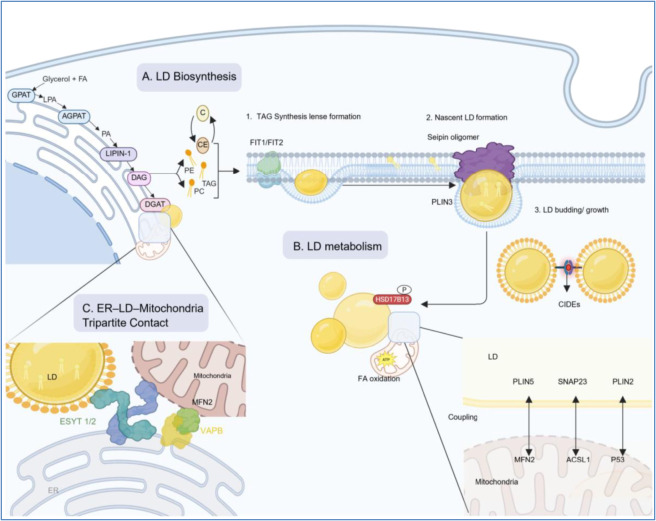
LD biogenesis, metabolism, and tripartite contacts. **(A)** LD Biogenesis. FAs are esterified to TAG, which with CE forms an oil lens budding from the ER. FIT1/2 and the seipin–LDAF1 complex promote nascent LD formation and cytosolic budding. Mature LDs subsequently expand through local TAG synthesis and CIDE-mediated lipid transfer. **(B)** LD metabolism. HSD17B13 localizes to the LD phospholipid monolayer, where phosphorylation at Ser33 can regulate ATGL–CGI-58 complex assembly and promote efficient lipolysis, thereby supporting fatty acid mobilization for mitochondrial β-oxidation. Efficient metabolic coupling between LDs and mitochondria is maintained by contact-site proteins, including PLIN5–MFN2, SNAP23–ACSL1, and PLIN2–p53, which coordinate lipid utilization with cellular energy demand. **(C)** ER–LD–mitochondria tripartite contact sites. The ER, LDs, and mitochondria establish specialized membrane contact sites that integrate lipid synthesis, lipid trafficking, and fatty acid oxidation. Representative tethering proteins, including ESYT1/2, VAPB, and MFN2, facilitate communication between these organelles, thereby maintaining hepatic lipid homeostasis. Disruption of these coordinated processes contributes to impaired fatty acid oxidation, lipid accumulation, and the progression of metabolic dysfunction-associated steatohepatitis (MASH).

LDs are highly dynamic organelles whose biogenesis, growth, inter-organelle contacts, and turnover are tightly regulated by a complex interplay of lipid metabolic enzymes, membrane-trafficking proteins, and regulatory cofactors (Henne and Cohen, 2026; Olzmann and Carvalho, 2019). Each LD consists of a hydrophobic core of TAG and sterol esters, surrounded by a phospholipid monolayer, a structural feature that distinguishes LDs from bilayer-enclosed organelles such as the ER, Golgi and mitochondria. This monolayer supports a specialized proteome of more than 100 proteins, including perilipins, CIDE family proteins and SDR enzymes such as HSD17B13 (Bersuker and Olzmann, 2017; Krahmer et al., 2013; Thiam et al., 2013). A typical 400-nm LD is estimated to contain approximately 21 million TAG molecules, corresponding to roughly 7 billion ATP equivalents (Mouskeftara et al., 2024). The monolayer is enriched in phosphatidylcholine, phosphatidylethanolamine and phosphatidylinositol, and its composition and biophysical properties critically influence both protein recruitment and the formation of organelle-contact sites (Chitraju et al., 2012) (Figure 3).

### 3.1 Lipid droplet biogenesis at the ER

LD biogenesis originates at the ER, where TAG is generated via the Kennedy pathway by sequential acyl transfer involving GPAT, AGPAT and DGAT1/2, while steryl esters are generated via ACAT1/2 (Petschnigg et al., 2009). Neutral lipid accumulation beyond ∼5–10 mol% triggers remixing into an intramembrane oil lens (Khandelia et al., 2010; Duelund et al., 2013). This transition is organized by several ER-resident proteins. The approximately 600 kDa seipin-LDAF1 complex nucleates LD and directs budding towards the cytosol; post-budding, LDAF1 dissociates from seipin and relocates to the LD surface (Salo et al., 2019; Schneiter and Choudhary, 2022; Zoni et al., 2021). FIT1/2 provide additional regulation, with FIT2 phosphatase activity towards phosphatidic acid contributing to ER membrane homeostasis and LD budding (Kadereit et al., 2008; Wang et al., 2022). Lipid composition, surface tension and the asymmetric distribution of these regulators favour LD emergence from the cytosolic leaflet (Chorlay and Thiam, 2018; Choudhary et al., 2015). Nascent lipid droplets remain physically connected to the endoplasmic reticulum via membrane stalks, enabling the exchange of neutral lipids, phospholipids, and lipid-droplet-associated proteins during lipid droplet growth and maturation, experimentally observed in yeast as 30–60 nm diameter structures (Wilfling et al., 2013). Reversible detachment involves the COPI coatomer, which is recruited to LDs by ARF1 and its guanine-nucleotide exchange factor GBF1. Transient COPI-dependent nanodroplet formation can restore membrane continuity and facilitate movement of integral membrane proteins, including DGAT2 and GPAT4, between the ER and LD (Wilfling et al., 2013). This bidirectional connectivity links LD biogenesis to ER homeostasis, explaining why disruption of either compartment affects the other.

### 3.2 Maturation, fusion and CIDE-mediated growth

Following nucleation, LDs expand through three complementary mechanisms. First, enzymes such as DGAT2 and GPAT4 that delocalize from the ER to the LD monolayer support local TAG synthesis (Wilfling et al., 2013). Second, persistent ER-LD bridges permit direct, non-vesicular transfer of neutral lipids. Third, LDs enlarge through CIDE-family-mediated lipid transfer and fusion at LD–LD contact sites (Gong et al., 2011). CIDEA, CIDEB and CIDEC/FSP27 anchor to LDs through C-terminal amphipathic helices, dimerize, and assemble into trans-organellar oligomers at apposed LD surfaces (Lyu et al., 2021; Wickner and Rizo, 2017). CIDEC depletion produces numerous small LDs, whereas CIDEC overexpression promotes LD enlargement; in humans, biallelic CIDEC/FSP27 loss-of-function is associated with fatty liver, severe insulin resistance, and hypertension, linking this pathway to metabolic liver disease (Rubio-Cabezas et al., 2009).

SNARE proteins also contribute to LD growth. The STX18-SNAP23-SEC22B complex localizes to LDs and interacts with CIDEC, and hepatic STX18 depletion reduces LD size in high-fat-diet-fed mice (Fu et al., 2023). Whether CIDE-dependent lipid-permeable junctions and SNARE zippering act sequentially or in parallel remains unresolved. HSD17B13 may additionally influence LD growth through a non-catalytic scaffolding function: by occupying monolayer surface area, it could alter the local density of CIDE assemblies and thereby modulate LD–LD lipid transfer. This proposed role is mechanistically distinct from the enzymatic activity discussed in Section 5.2 (Thiam et al., 2013).

### 3.3 Catabolism by lipolysis and lipophagy

LD catabolism is accomplished via two principal routes: cytosolic lipolysis and autophagic degradation (lipophagy). Cytosolic lipolysis is initiated by adipose triglyceride lipase (ATGL), which hydrolyses TAG to diacylglycerol; hormone-sensitive lipase and monoacylglycerol lipase then subsequently release the remaining fatty acids. ATGL activity is dependent on its coactivator CGI-58/ABHD5, whose availability at the LD surface is governed by perilipins, particularly PLIN1 and PLIN5 in oxidative tissues, and potentially by HSD17B13 in hepatocytes (Su et al., 2022; Wilfling et al., 2013). In the liver, ATGL-derived fatty acids support both mitochondrial β-oxidation and VLDL assembly. Disruption of this lipolytic pathway-whether loss of ATGL/CGI-58 activity, accumulation of the PNPLA3 p. I148M variant or altered HSD17B13 Ser33 phosphorylation function can promote hepatic steatosis (Friedman et al., 2018).

Lipophagy provides a second route for LD turnover by delivering LD fragments to lysosomes for degradation. This process is activated during nutrient deprivation via AMPK and suppressed during nutrient excess through mTORC1 (Singh et al., 2009). Lipophagy and mitophagy share overlapping autophagic machinery and are functionally interdependent; impairment of either process can therefore compromise cross-organelle quality control and favor the progression from simple steatosis to MASH. Additionally, chaperone-mediated autophagy of LD-associated perilipins PLIN2 and PLIN3 by Hsc70 is required to expose the LD surface to lipases and the macroautophagic machinery. Failure to remove these perilipins restricts LD catabolism and promotes lipid accumulation (Roberts and Olzmann, 2020; Schott et al., 2019).

### 3.4 The LD proteome and its broader significance

The LD proteome is highly dynamic. Its composition changes with metabolic state, differs between TAG-rich and steryl-ester-rich LDs, and extends well beyond proteins involved directly in lipid storage. PLIN2 and PLIN3 are abundant on hepatocyte LDs and are increased in MASLD liver samples (Ma et al., 2024). Inflammatory stimuli can remodel this surface: bacterial lipopolysaccharide enriches innate immune proteins on LDs while displacing PLIN5, thereby weakening LD-mitochondria coupling and shifting metabolism away from oxidative phosphorylation (Bosch et al., 2020). LDs also store arachidonic acid and recruit phospholipases, cyclooxygenases and lipoxygenases, positioning them at the centre of eicosanoid production and inflammatory resolution (Jarc and Petan, 2020). Thus, in the context of liver disease, LDs function not only as energy stores but also as signalling platforms that coordinate lipid-derived inflammatory responses.

This convergence of energy storage, inflammatory signalling, and mitochondrial contact transforms the LD monolayer into an active regulatory surface. Among its resident proteins, HSD17B13 is distinguished by the strength of the associated human genetic associations and by its potential to couple LD structure to lipid flux. These properties position HSD17B13 as a key node within the LD-mitochondria axis, warranting the detailed mechanistic consideration that follows in the subsequent sections.

## 4 The LD-mitochondria contact site interface in MASH

The central premise of this review is that MASH involves dysfunction not only within LDs and mitochondria but also at the interface that coordinates them. LD-mitochondria contact sites (LDMCs) are regulated structures that facilitate the direct transfer of lipolysis-derived fatty acids into mitochondrial oxidative pathways, limit the accumulation of unesterified fatty acids in the cytosol and adapt substrate delivery to nutritional state (Benador et al., 2018; Benador et al., 2019). Loss or remodeling of these contacts may therefore contribute to the transition from steatosis to steatohepatitis and represents a potential therapeutic target.

### 4.1 Physical tethers

Multiple molecular complexes contribute to LD-mitochondria tethering in mammalian cells, and current evidence suggests that they define functionally distinct contact-site populations (Miner et al., 2023). PLIN5-MFN2 is the best-characterized hepatic module. PLIN5 is enriched in oxidative tissues and recruit mitochondria to LDs through its C-terminal region; its association with MFN2, potentially involving Hsc70-dependent intermediates, forms a bridge that coordinates fatty-acid release to β-oxidation (Wang et al., 2011; Talari et al., 2023; Fan and Tan, 2024). The metabolic consequence of this interaction depends on PLIN5 phosphorylation. Phosphorylation-deficient PLIN5 favours persistent LD-mitochondria association and TAG storage, whereas phosphomimetic PLIN5 facilitates fatty-acid mobilization and oxidation (Segales and Liesa, 2026). Thus, the same contact module can support either storage or oxidation depending on the hormonal and nutritional context.

VPS13D-TSG101 defines a parallel contact-site pathway that is required for starvation-induced fatty-acid transfer when PLIN5-dependent tethering is absent (Wang et al., 2021). VPS13D associates with LDs through C-terminal amphipathic helices and with mitochondria through its N-terminal region. It also recruits ESCRT components, including CHMP6, CHMP4B, CHMP1B, IST1 and ALIX, which may support the membrane remodelling required for lipid transfer (Vietri et al., 2020). Depletion of these ESCRT proteins impairs LD-to-mitochondria fatty-acid transport, supporting a model in which VPS13D coordinates tethering with local membrane dynamics.

A third module involves ACSL1 and SNAP23. ACSL1 activates fatty acids to acyl-CoA and interacts with LD-associated SNAP23, thereby coupling organelle apposition to metabolic channeling of activated fatty acids (Young et al., 2018; Jagerstrom et al., 2009). Additional contact proteins include MIGA2, which links mitochondria to both LDs and the ER in adipocytes through VAPA/VAPB, and components of the mitochondrial TOM/SAM/MIM import machinery, which can provide structural support for organelle association (Freyre et al., 2019; Becker et al., 2011; Hulett et al., 2008; Drwesh and Rapaport, 2020). The relative contribution of these systems in hepatocytes, and their degree of redundancy during MASH progression, remains incompletely defined.

Collectively, these observations suggest that MASH is accompanied by coordinated disruption of multiple tethering pathways, including acetylation-dependent loss of MFN2 (Joaquim et al., 2025), altered PLIN5 phosphorylation (Segales and Liesa, 2026) and inflammation-induced remodelling of the LD proteome (Bosch et al., 2020). Therapeutic strategies may therefore need to restore contact-site function at the network level rather than target a single tether. In this context, HSD17B13 modulation, AMPK activation, and correction of mitochondrial dynamics represent conceptually distinct but potentially complementary approaches to this objective.

### 4.2 Functional consequences from physiological channelling to MASH decoupling

Under physiological conditions, LDMCs establish an efficient route for substrate transfer between storage and oxidation. During fasting or β-adrenergic stimulation, regulated tethering and lipolysis support β-oxidation and ketogenesis while limiting the cytosolic accumulation of potentially toxic free fatty acids. The LipoID proximity-labelling approach has further uncovered VDAC3-PLIN3 as a functional contact module whose abundance is sensitive to mitochondrial membrane potential and ATP availability (Guo et al., 2026). Pharmacological inhibition of VDAC3 or dissipation of membrane potential reduces LD-mitochondria proximity and alters cellular lipid composition, indicating that mitochondrial energetic state can directly influence contact-site architecture.

In MASH, this coupling is disrupted at multiple levels. Degradation or displacement of tethering proteins reduces contact-site abundance; DRP1-dependent fragmentation generates smaller mitochondrial units that are less capable of sustaining stable LD contacts (Sassano et al., 2017); altered PLIN5 phosphorylation can favour storage over oxidation (Segales and Liesa, 2026); and inflammatory remodeling of the LD proteome further displaces coupling factors (Bosch et al., 2020). The resulting combination of enlarged LDs, fragmented mitochondria, diminished organelle coupling, and impaired ATP production represents a hallmark cellular phenotype of progressive liver disease.

### 4.3 The tripartite ER-LD-mitochondria hub

Mitochondria-ER contact sites, maintained at a separation of approximately 10–30 nm, regulate calcium flux, lipid metabolism, redox signaling and apoptosis (Rui, 2014). LDs participate in tripartite contacts with both the ER and mitochondria through proteins such as ESYT1/2 and VAPB; deletion of these factors enlarges LDs and compromises mitochondrial β-oxidation (Hung et al., 2014). GBF1, the ARF1 guanine-nucleotide exchange factor involved in LD detachment from the ER, also interacts with MIRO to influence mitochondrial positioning and cristae architecture, and regulates ATGL delivery to LDs (Ellong et al., 2011; Walch et al., 2018). Collectively, these findings support the view that the ER, LDs and mitochondria form an integrated metabolic continuum, within which specialized contact-site populations may support FAO, VLDL assembly or ketogenesis and may be differentially affected in MASH.

An unresolved question is whether individual tethering complexes can be stabilized directly in diseased hepatocytes, or whether improvements in contact-site organization will arise primarily as secondary consequences of upstream interventions, such as HSD17B13 modulation, AMPK activation or GLP-1 receptor agonism. Supporting evidence exists for both scenarios, but remains insufficient to establish a causal hierarchy. This uncertainty provides an important context for evaluating HSD17B13 as a regulator of LD function and organelle coupling.

## 5 HSD17B13 as an important regulator of the LD-mitochondria communication

**FIGURE 4:**
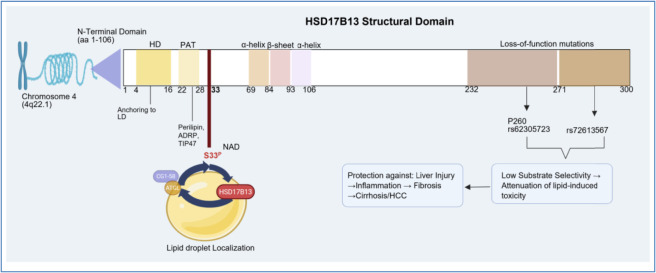
Structural organisation and disease-associated variants of HSD17B13. HSD17B13 contains an N-terminal hydrophobic LD-anchoring region, a PAT-like motif and an SDR catalytic core with a Rossmann fold for dinucleotide binding. PKA-dependent phosphorylation at Ser33 regulates coupling to the CGI-58/ATGL lipolytic machinery. Loss-of-function variants, including rs72613567: TA and variants affecting the C-terminal region, are associated with reduced risk of progressive chronic liver disease. Domain boundaries and functional assignments shown in the schematic should be interpreted in the context of the available structural and biochemical evidence.

If impaired LD-mitochondria communication contributes to MASH, HSD17B13 represents a compelling candidate regulatory node. However, a key distinction must be made between structural organelle tethers and functional metabolic coupling. While direct visual or biochemical evidence of HSD17B13 acting as a physical bridge at lipid droplet–mitochondria contact sites (LDMCs) is currently lacking, human genetics and mechanistic models provide strong rationale for its role as a metabolic switch gating inter-organelle lipid flux. The definitive evidence for this functional coupling relies on the finding that phosphorylation of HSD17B13 at serine 33 (Ser33) promotes the functional assembly of the ATGL–CGI-58 lipolytic complex at the LD surface. In HSD17B13-S33A knock-in mice, disruption of this phosphorylation restricts triacylglycerol hydrolysis, directly compromises downstream mitochondrial β-oxidation, and exacerbates hepatic steatosis and inflammation. Conversely, β-adrenergic stimulation restores mitochondrial substrate disposal and ameliorates steatohepatitis. These findings establish HSD17B13 as a dynamic regulatory valve that aligns lipid droplet turnover with mitochondrial energy demand, offering a clear mechanism for the organelle uncoupling observed in MASH. The following sections review the structural and functional evidence for HSD17B13 and distinguish established findings from unresolved mechanistic questions that bear directly on therapeutic development (Figure 4).

### 5.1 Discovery, hepatocyte-specific LD localization and structural architecture

Identified in 2007 as SCDR9, HSD17B13 shares approximately 67% amino acid sequence identity with HSD17B11 within the conserved catalytic domain, supporting their evolutionary relationship through gene duplication (Liu et al., 2023; Horiguchi et al., 2008). The HSD17B13 gene is located on chromosome 4q22.1 and exhibits considerable transcript diversity as a result of alternative splicing. While eight distinct exonic regions are identified across all annotated transcript isoforms, individual HSD17B13 transcripts typically comprise six or seven exons. HSD17B13 is located adjacent to HSD17B11; however, because both genes are encoded on the negative DNA strand, HSD17B13 lies approximately 13 kb downstream of HSD17B11 in the direction of transcription (Zhang et al., 2022). This distinction between transcriptional orientation and physical genomic coordinates is important to avoid confusion when describing the genomic organization of the HSD17B13 locus (Liu et al., 2023; Horiguchi et al., 2008).

HSD17B13 expression is strongly enriched in the liver, with weak expression reported in the ovary, kidney, brain, lung, skeletal muscle and bladder. Like other SDR enzymes, HSD17B13 contains a Rossmann-fold TGXGXXXG motif for NAD(P)^+^/NAD(P)H binding and a YXXXK catalytic motif (Liu et al., 2023; Horiguchi et al., 2008). Subcellular localization is a defining feature of HSD17B13. In Huh7 cells, HSD17B13–GFP localizes predominantly to the LD monolayer, with little detectable signal in the ER, mitochondria or cytosol (Su et al., 2014). The N-terminal residues 1–28 form a hydrophobic segment that inserts into the monolayer, and Pro28 is important for LD targeting (Horiguchi et al., 2008). The catalytic core extends approximately from residues 29 to 259, whereas residues 260–286 form an amphipathic helix-turn-helix that further stabilizes monolayer association, with Pro260 and Pro274 marking structural boundaries (Liu et al., 2023). HSD17B13 forms a stable dimer whose interface buries more than 2,000 Å^2^ per monomer and includes residues required for catalysis, thereby coupling oligomerization to enzymatic competence (Liu et al., 2023). Structural models show a sled-like orientation, with the N-terminal hydrophobic helices inserted into the monolayer and the C-terminal amphipathic helices interacting with phospholipid headgroups. This arrangement provides a plausible explanation for preferential localization to LD monolayers rather than ER bilayers (Liu et al., 2023).

An α/β/α structural element spanning residues 69–106 is required for correct folding and ER-to-LD trafficking; deletion of this region causes ER retention and proteasomal degradation (Liu et al., 2023; Horiguchi et al., 2008). Comparative studies of human and canine HSD17B13 suggest that both the ligand-binding and membrane-anchoring regions are conformationally dynamic, potentially allowing transient access of lipid substrates to the catalytic pocket. Species-specific residues, including E177/G178/A205 in human HSD17B13 and G177/V178/T205 in the canine protein, influence substrate selectivity and complicate extrapolation across experimental models (Liu et al., 2023; Inderbinen et al., 2020). Despite extensive biochemical analysis, the physiological substrate remains uncertain. Recombinant assays have implicated steroids, leukotrienes B3/B4, retinol and selected phospholipids, but none has been established unequivocally as the dominant substrate in hepatocytes or *in vivo* (Abul-Husn et al., 2018; Ma et al., 2019).

### 5.2 Proposed dual function of HSD17B13 as a lipogenic and lipolytic switch

HSD17B13 has been proposed to serve two mechanistically distinct functions at the LD surface. Its lipogenic role is linked to SREBP-1c and LXRα, which induce HSD17B13 expression as part of the hepatic lipogenic signaling; HSD17B13 may in turn facilitate SREBP-1c maturation, thereby establishing a positive-feedback circuit that promotes LD expansion under nutrient excess (Su et al., 2019). In Huh7 cells, HSD17B13 overexpression increases intracellular TAG content, and these effects require both LD targeting and an intact catalytic domain (Su et al., 2014; Adam et al., 2018). Whether this phenotype reflects enzymatic activity, a scaffolding function or both remains an important unresolved question.

A second proposed function involves regulated lipolysis through phosphorylation of HSD17B13-Ser33, an evolutionarily conserved residue within the N-terminal LD-targeting region. PKA-mediated Ser33 phosphorylation promotes functional coupling between ATGL and its coactivator CGI-58/ABHD5 at the LD surface (Su et al., 2022). In HSD17B13-S33A knock-in mice, loss of this phosphorylation site reduces ATGL-dependent lipolysis, compromises mitochondrial function and promotes hepatic steatosis and inflammation (Su et al., 2022). The β-adrenergic agonist reproterol increases Ser33 phosphorylation and ATGL activity and ameliorates diet-induced hepatic steatosis in mice while suppressing SREBP-1c signaling (Su et al., 2022). These observations identify phosphorylation-dependent regulation of HSD17B13 as a potential link between hormonal signaling, LD turnover and mitochondrial substrate supply.

Collectively, these findings suggest that HSD17B13 may integrate lipogenesis with lipolysis. This duality could help explain why both increased expression and complete loss of HSD17B13 produce context-dependent phenotypes in different experimental systems. It also cautions against treating HSD17B13 as a conventional metabolic enzyme whose function can be inferred solely from catalytic assays.

### 5.3 Human genetics of HSD17B13 in MASLD and related liver diseases

The exome-wide association study by Abul-Husn and colleagues identified HSD17B13 rs72613567:TA, an adenine insertion at the donor splice site of exon 6, as a common loss-of-function variant (Abul-Husn et al., 2018). The insertion disrupts normal splicing and generates a truncated, unstable protein with markedly reduced enzymatic activity. In population studies, the variant has been associated with lower circulating aminotransferase concentrations and reduced risk of chronic liver disease, including cirrhosis and alcohol-related liver disease; an association with lower HCC risk has also been reported (Abul-Husn et al., 2018). Protective associations have subsequently been observed in Chinese Han and other populations, although effect sizes vary across ancestry and clinical context (Pirola et al., 2019; Chen et al., 2020).

The genetic architecture of MASLD is polygenic, and the effect of HSD17B13 must therefore be interpreted alongside other risk loci. Combined scores incorporating PNPLA3 p. I148M, TM6SF2 p. E167K and HSD17B13 variants improve risk stratification for cirrhosis and HCC (Gellert-Kristensen et al., 2020; Kozlitina, 2020), and broader models that include MBOAT7 rs641738 and clinical variables may further improve prediction of MASH severity and advanced fibrosis (Bianco et al., 2021; Paternostro et al., 2021). The protective association of rs72613567: TA also appears to vary with age, sex, obesity, diabetes and PNPLA3 genotype (Vilar-Gomez et al., 2021). Mechanistically, PNPLA3 p. I148M accumulates on LDs and interferes with ATGL–CGI-58-dependent lipolysis; HSD17B13 loss-of-function could modify this phenotype by altering competition for LD-surface scaffolds, although this interaction remains to be established directly.

Additional HSD17B13 alleles, including rs62305723, rs6834314, rs9992651, rs13118664 and rs143404524, have been associated with altered splicing or amino-acid substitutions within LD-targeting or catalytic regions (Anstee et al., 2020). The convergence of independent loss-of-function alleles on a broadly protective clinical phenotype provides strong genetic support for therapeutic inhibition. Nevertheless, the extent to which partial catalytic inhibition, protein depletion and complete genetic loss are biologically equivalent remains uncertain and requires direct clinical evaluation.

### 5.4 Mouse models and species discordance

Despite the consistency of the human genetic signal, phenotypes in mouse models are heterogeneous. Some studies report that mouse Hsd17b13 deletion reduces diet-induced steatohepatitis, fibrosis and hepatocyte injury (Adam et al., 2018; Luukkonen et al., 2023). Lipidomic analyses have identified changes in hepatic phosphatidylcholine, diacylglycerol, ceramide and retinoid metabolism that could influence lipotoxicity and HSC activation (Su et al., 2017). Other work has linked HSD17B13 deficiency to altered expression or activity of phosphatidylcholine-synthesis enzymes, including CEPT1 and PEMT, suggesting that phospholipid homeostasis may contribute to the phenotype (Zhang et al., 2025).

Other studies, however, report little protection or spontaneous steatosis after Hsd17b13deletion under different dietary or genetic conditions. Several factors may contribute to these discrepancies, including species-specific substrate selectivity, compensatory expression of paralogues such as HSD17B11, and strain- or diet-dependent regulation of the SREBP-1c–HSD17B13 axis. Humanized systems such as hepatocyte-specific expression of human HSD17B13 on an Hsd17b13-null background or models carrying the rs72613567: TA allele in a humanized liver will be important for establishing pharmacological relevance and improving translation.

### 5.5 Multilayered regulation of HSD17B13 expression and activity

HSD17B13 is regulated at several levels. Transcription is driven in part by the SREBP-1c/LXRα axis (Su et al., 2017; Beaven et al., 2011), while post-transcriptional control may involve microRNAs and RNA-binding proteins responsive to metabolic, inflammatory and ER-stress signals. PKA-dependent phosphorylation of Ser33 is the best-characterized post-translational mechanism, and ubiquitination may further influence protein stability and LD-surface abundance. Thus, HSD17B13 activity reflects the combined effects of lipogenic transcription, hormonal cAMP signalling and protein turnover, providing multiple potential points for pharmacological modulation.

## 6 From genetic protection to first-in-class therapeutics

**FIGURE 5:**
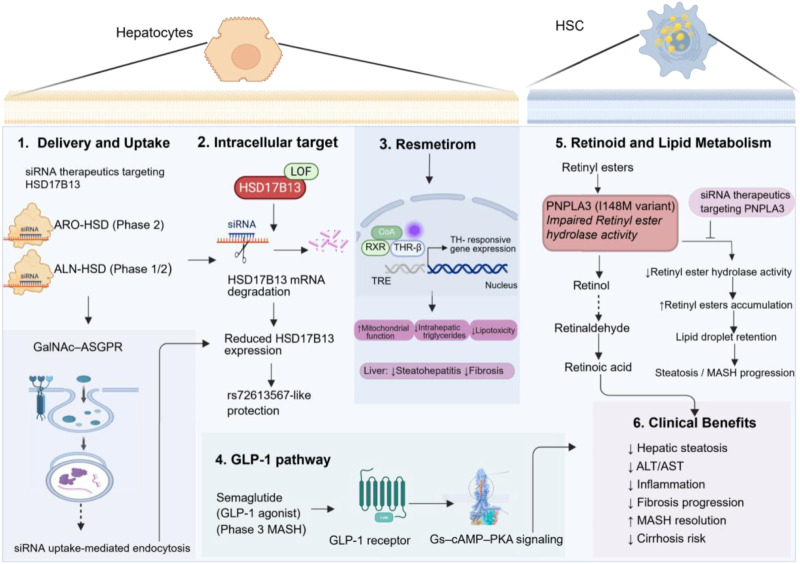
Therapeutic strategies converging on the LD-mitochondria axis. (1) GalNAc-conjugated siRNAs targeting HSD17B13 reduce hepatic HSD17B13 expression and are designed to mimic the protective loss-of-function phenotype. (2) Resmetirom activates THRβ-dependent transcriptional programmes that enhance mitochondrial lipid oxidation and improve lipid homeostasis. (3) Retinoid metabolism and PNPLA3 p. I148M provide additional LD-associated pathways. (4) Semaglutide acts primarily through systemic GLP-1 receptor-dependent mechanisms. (5) These interventions converge on reductions in hepatic lipid accumulation, inflammation and fibrosis. Combination strategies should be evaluated according to mechanistic complementarity, genotype and disease stage.

The protective association of HSD17B13 loss-of-function variants has stimulated the development of two principal therapeutic modalities: hepatocyte-targeted RNA interference and selective small-molecule inhibition. Early clinical investigations are evaluating whether pharmacological suppression can reproduce the biochemical and histological features associated with the human genetic phenotype. This section reviews these approaches, places them within the broader therapeutic landscape of MASH and considers the rationale for mechanism-based combination therapy (Figure 5; Table 1).

**TABLE 1:** Therapeutic strategies converging on the lipid droplet–mitochondria axis in MASH.

Strategy	Representative agent(s)	Target/modality	Mechanistic action	Clinical and translational positioning
HSD17B13-directed therapies				
RNA interference	GSK4532990 (ARO-HSD); ALN-HSD	GalNAc-siRNA; HSD17B13 mRNA	Hepatocyte-selective knockdown reduces HSD17B13 transcript and protein, thereby mimicking protective loss-of-function alleles.	Early clinical development; provides the most direct test of human genetic target validation (Mak et al., 2023; Sanyal et al., 2025; Thamm et al., 2023)
Small-molecule inhibition	BI-3231; INI-822	HSD17B13 catalytic site	Selective enzymatic inhibition may distinguish catalytic activity from the protein’s lipid droplet scaffolding function.	BI-3231 is a preclinical chemical probe; INI-822 is in Phase I evaluation (Thamm et al., 2023; Liu et al., 2007)
Mechanistically complementary therapies				
THR-β agonism	Resmetirom	THR-β	Enhances mitochondrial fatty-acid oxidation and biogenesis, suppresses *de novo* lipogenesis and improves cholesterol handling.	Approved MASH therapy; a rational partner for restoring use of fatty acids released from lipid droplets (Harr et al., 2019; Kannt et al., 2021)
GLP-1 receptor agonism	Semaglutide	GLP-1R; systemic metabolic control	Improves body weight, insulin sensitivity and systemic substrate flux, with predominantly indirect hepatic effects.	Advanced clinical development; complements hepatocyte-selective HSD17B13 targeting (Newsome et al., 2021; Michel and Schattenberg, 2025)
SCD1 modulation	Aramchol	SCD1	Reduces *de novo* lipogenesis while modulating inflammatory and fibrogenic pathways.	May reduce lipid input while HSD17B13-directed therapy improves lipid droplet turnover (Ratziu et al., 2021)
PPAR agonism	Saroglitazar	PPAR-α/γ	Promotes fatty-acid oxidation and improves insulin sensitivity and atherogenic dyslipidaemia.	Clinical-stage metabolic complement to HSD17B13-directed therapy (Gawrieh et al., 2021)
PDE inhibition	ZSP1601	Pan-PDE	Modulates cyclic-nucleotide signalling and has reduced liver fat and injury markers in early studies.	Early clinical development; combination value remains to be established (Hu et al., 2023)
FXR agonism	Obeticholic acid	FXR	Modulates bile-acid signalling, lipid metabolism, inflammation and fibrogenic pathways.	Histological activity has been tempered by tolerability and regulatory limitations (Anstee et al., 2020; Loomba and Sanyal, 2013)
Genotype-directed RNA interference	ARO-PNPLA3 (JNJ-75220795)	GalNAc-siRNA; PNPLA3 I148M	Reduces pathogenic PNPLA3 accumulation on lipid droplets and may relieve interference with ATGL–CGI-58-mediated lipolysis.	A genotype-defined partner for patients carrying PNPLA3 risk alleles (Jeon et al., 2025)

Abbreviations: ATGL, adipose triglyceride lipase; DNL, *de novo* lipogenesis; FAO, fatty-acid oxidation; FXR, farnesoid X receptor; LD, lipid droplet; PDE, phosphodiesterase; PPAR, peroxisome proliferator-activated receptor; THRβ, thyroid hormone receptor β. Clinical status is presented as reported in the cited studies and in the manuscript.

### 6.1 RNA interference targeting HSD17B13

Clinical RNAi strategies targeting HSD17B13 use GalNAc conjugation to achieve hepatocyte-selective delivery via the asialoglycoprotein receptor (ASGPR). After receptor-mediated endocytosis and intracellular release, the siRNA guide strand is incorporated into the RNA-induced silencing complex and directs Argonaute-2-dependent cleavage of *HSD17B13* mRNA (Springer and Dowdy, 2018; Alshaer et al., 2021). This delivery platform has been validated across multiple liver-directed RNAi therapeutics and supports durable target suppression with limited extrahepatic exposure.

GSK4532990 (formerly ARO-HSD) is a double-stranded GalNAc-conjugated RNAi therapeutic originally developed by Arrowhead Pharmaceuticals and subsequently licensed to GSK (Mak et al., 2023). Early clinical data showed dose-dependent reduction in hepatic HSD17B13 mRNA and protein, together with decreases in serum ALT and AST. These findings established pharmacological target engagement and supported further evaluation in patients with MASH.

ALN-HSD, developed by Alnylam Pharmaceuticals and Regeneron, is administered subcutaneously and similarly uses GalNAc-mediated hepatocyte targeting *HSD17B13* mRNA. In the Phase I study (NCT04565717), ALN-HSD was generally well tolerated in healthy participants and patients with MASH. In 46 patients with MASH randomised to receive 25, 200, or 400 mg of ALN-HSD or placebo on days 1 and 85, the drug produced dose-dependent reductions in hepatic *HSD17B13* mRNA. Mild and transient injection-site reactions were the only adverse event occurring in at least 10% of participants, and no treatment-emergent adverse events of clinical concern were reported. Reduced hepatic HSD17B13 mRNA relative to placebo and was associated with exploratory improvements in the NAFLD activity score (Sanyal et al., 2025). Ongoing studies will be important for determining whether sustained gene silencing translates into reproducible histological benefit and an acceptable long-term safety profile.

### 6.2 Small-molecule inhibitors of HSD17B13

Small-molecule inhibitors provide a complementary strategy that may permit oral dosing, shorter pharmacological persistence and selective interrogation of catalytic function. BI-3231, also reported as compound 45, was developed through high-throughput screening and medicinal-chemistry optimization and is widely used as a selective HSD17B13 chemical probe (Thamm et al., 2023). Its binding and inhibitory activity are NAD-dependent, and preclinical studies indicate preferential hepatic exposure. BI-3231 has been used to examine the consequences of catalytic inhibition on hepatocyte TAG accumulation, mitochondrial respiration and cell-state phenotypes (Thamm et al., 2023). The scaffold may also provide a starting point for proteolysis-targeting chimaeras, allowing comparison of catalytic inhibition with complete protein degradation and thereby helping to distinguish enzymatic from scaffolding functions.

INI-822 is a selective small-molecule HSD17B13 inhibitor undergoing early clinical evaluation for MASH (NCT05945537) (Liu et al., 2007). Preliminary reports describe reductions in alanine aminotransferase and changes in hepatic phosphatidylcholine and putative HSD17B13 lipid substrates. Although these observations are consistent with target engagement, peer-reviewed clinical data will be required to establish their magnitude, durability and relationship to histological outcomes.

Structural information remains a major limitation for drug development. A high-resolution structure of full-length, membrane-associated HSD17B13 in complex with an inhibitor has not yet been reported. Current medicinal-chemistry efforts therefore rely partly on modelling from HSD17B11, whose available structure lacks the LD-targeting helices and the complete membrane context (Horiguchi et al., 2008). Structural determination of full-length HSD17B13 in nanodiscs or other LD-mimetic systems could clarify substrate access, cofactor dependence and inhibitor binding and would provide a stronger basis for structure-guided optimization.

### 6.3 Combinatorial strategies for HSD17B13 with nuclear receptor and incretin co-targeting

MASH arises from interacting hepatic and systemic abnormalities and is therefore unlikely to be controlled by modulation of a single pathway. HSD17B13-directed therapy primarily targets LD-associated lipid handling, whereas complementary agents can address mitochondrial dynamics, bile-acid signalling, insulin resistance, body weight and fibrosis (Table 1).

Resmetirom (MGL-3196) was approved by the United State Food and Drug Administration in March 2024 for adults with non-cirrhotic MASH and moderate-to-advanced fibrosis (FDA Approved). This liver-directed thyroid hormone receptor-β (THRβ) agonist promotes transcriptional programmes that increase mitochondrial fatty-acid oxidation, improve cholesterol handling and suppress lipogenic pathways (Harr et al., 2019; Kannt et al., 2021). In the MAESTRO-NASH Phase III trial, resmetirom increased the proportion of patients achieving MASH resolution without worsening fibrosis and fibrosis improvement without worsening MASH at 52 weeks (FDA Approved). Mechanistically, resmetirom may complement HSD17B13 inhibition by increasing oxidative capacity while HSD17B13-directed therapy modifies fatty-acid mobilisation and LD-surface regulation.

Semaglutide, a long-acting GLP-1 receptor agonist, improves body weight, insulin sensitivity and several histological features of MASH (Newsome et al., 2021; Michel and Schattenberg, 2025). Because GLP-1 receptor expression in hepatocytes is low, its hepatic effects are thought to arise predominantly through extrahepatic actions involving the pancreas, central nervous system, gastrointestinal tract and cardiovascular system (Drucker, 2018; Holst et al., 2020). Combining semaglutide with hepatocyte-directed HSD17B13 suppression is therefore mechanistically attractive: semaglutide addresses systemic metabolic drivers, whereas HSD17B13-directed therapy acts more directly on hepatic LD biology and lipid flux.

Several additional agents target complementary pathways. Aramchol partially inhibits stearoyl-CoA desaturase 1 and modulates lipogenesis, inflammation and fibrosis (Ratziu et al., 2021). Saroglitazar, a dual PPARα/γ agonist, improves liver enzymes, hepatic fat and atherogenic dyslipidaemia in MASLD/MASH (Gawrieh et al., 2021). ZSP1601, a pan-phosphodiesterase inhibitor, has shown early signals of reduced liver fat and fibrosis-associated biomarkers (Hu et al., 2023). FXR agonists, including obeticholic acid, modulate bile-acid and fibrogenic pathways but have been limited by adverse effects and regulatory uncertainty (Anstee et al., 2020; Loomba and Sanyal, 2013). PNPLA3-targeted RNAi, including ARO-PNPLA3/JNJ-75220795, provides another genetically informed strategy for patients carrying PNPLA3 p. I148M (Jeon et al., 2025). Together, these programmes illustrate the expanding range of pathways that could be combined with HSD17B13-directed therapy.

The rationale for combination therapy is therefore based on mechanistic complementarity. HSD17B13 suppression may modify LD-surface regulation and lipid flux; THRβ, PPAR and FXR agonists reprogramme oxidative, lipogenic and bile-acid pathways; GLP-1 receptor agonists address obesity and systemic insulin resistance; and SCD1 or ACC inhibitors reduce DNL. Genotype-informed trials—such as testing HSD17B13 inhibition with resmetirom in PNPLA3 p. I148M carriers who lack protective HSD17B13 alleles—could improve both biological interpretation and identification of responsive patient subgroups.

### 6.4 Remaining needs for HSD17B13-directed therapy

Several gaps must be addressed before HSD17B13-directed therapy can be positioned clinically. First, validated pharmacodynamic biomarkers of HSD17B13 activity are lacking, complicating dose selection and confirmation of target engagement; candidate approaches include circulating exosomal HSD17B13 and lipid-substrate signatures, but neither has been validated. Second, HSD17B13, PNPLA3 and TM6SF2 genotypes have not yet been incorporated systematically into trial design, despite their potential to influence treatment response. Third, comparative data against established and emerging therapies, including resmetirom, are absent. Ongoing clinical studies will therefore determine whether HSD17B13 inhibition is most effective as monotherapy in selected patients or as a component of combination regimens.

## 7 Unresolved questions and future directions

The evidence reviewed here supports a model in which MASH involves defective communication between LDs and mitochondria in addition to abnormalities within either organelle alone. HSD17B13 is particularly relevant because it combines strong human genetic support with localization to the LD surface and plausible roles in both lipid metabolism and organelle coupling. Translating this framework into therapy, however, requires resolution of several fundamental questions.

### 7.1 The unresolved substrate questions

Despite extensive biochemical study, the endogenous substrate or substrate class that is most relevant to HSD17B13 function in human liver remains unknown. Recombinant assays have identified activity towards steroids, leukotrienes B3/B4, retinol and selected phospholipids, but none have been linked conclusively to the protective phenotype of rs72613567: TA carriers (Abul-Husn et al., 2018; Kozlitina et al., 2018). Reduced circulating IL-6 in loss-of-function carriers is compatible with altered inflammatory lipid metabolism (Kozlitina et al., 2018), whereas lipidomic studies in Hsd17b13-deficient mice implicate phosphatidylcholine, ceramide and retinoid pathways (Su et al., 2017). Resolving this question will require genotype-stratified lipidomics in human liver, integrated with isotope tracing and pharmacological perturbation in primary hepatocytes or organoid systems. Identification of a disease-relevant substrate would also enable direct pharmacodynamic biomarker development.

### 7.2 Scaffold versus enzymatic activity: which function to target?

A second question is whether MASH-associated phenotypes depend predominantly on the catalytic activity of HSD17B13 or on non-enzymatic functions at the LD surface. The rs72613567: TA variant reduces both because the resulting protein is truncated and unstable. By contrast, siRNA-based agents deplete the protein, whereas small-molecule inhibitors primarily suppress catalysis and may preserve scaffolding interactions. Comparative clinical and translational analyses of these modalities could therefore provide a natural experiment. Protein degraders derived from BI-3231-like scaffolds would offer an additional approach by separating inhibition from complete removal (Thamm et al., 2023). Resolving this distinction is essential for defining the optimal therapeutic modality and may provide a broader precedent for other multifunctional LD proteins.

### 7.3 Contact-site heterogeneity as a stratification axis

LD-mitochondria contacts are heterogeneous. PLIN5-MFN2, VPS13D-TSG101, ACSL1-SNAP23 and other modules appear to occupy distinct cellular contexts and may support different functions, including FAO, lipid synthesis and ketogenesis (Wang et al., 2021; Young et al., 2018; Miner et al., 2023). This heterogeneity is not captured by current clinical assessments but could influence therapeutic response. Patients with predominant loss of oxidation-coupled contacts might benefit most from interventions that improve tethering or mitochondrial capacity, whereas patients with persistent synthesis-oriented contacts might require stronger suppression of DNL. Candidate stratification approaches include biopsy-based proximity assays, imaging of fatty-acid flux and circulating markers of tether turnover. Establishing the biological specificity and clinical feasibility of such measures is an important medium-term objective.

### 7.4 A unifying model and translational outlook

The available evidence can be integrated into a four-component model of MASH progression. First, defects in mitochondrial dynamics and quality control generate fragmented, ROS-producing organelles. Second, dysregulated LD biogenesis and turnover increase the pool of lipotoxic lipid species. Third, loss or reprogramming of LD-mitochondria contacts uncouple lipid release from oxidative disposal. Fourth, mtDNA release and inflammatory signalling through cGAS–STING, NLRP3 and HSC pathways amplify tissue injury and fibrosis. These components form a self-reinforcing network rather than a simple linear cascade. HSD17B13 lies at the LD-facing portion of this network and may influence both lipid mobilization and the organization of the LD surface, making it a strategically positioned—but not necessarily singular—therapeutic node.

This model suggests that HSD17B13 inhibition may be most effective as part of a mechanism-based combination regimen, particularly in patients with advanced or metabolically complex disease. Potential combinations include HSD17B13 inhibition with resmetirom to align LD-derived substrate supply with increased oxidative capacity, with semaglutide to pair hepatocyte-directed action with systemic metabolic improvement, or with PNPLA3-targeted RNAi to address complementary LD-associated genetic mechanisms. Prospective stratification by HSD17B13, PNPLA3 and TM6SF2 genotype would strengthen biological interpretation and may help identify treatment-responsive subgroups.

Progress in four areas would substantially accelerate this field. High-resolution structures of full-length, membrane-associated HSD17B13 would support rational inhibitor and degrader design. Biomarkers of LD–mitochondria contact integrity and HSD17B13 activity would improve dose selection and patient stratification. Humanized experimental models carrying clinically relevant HSD17B13 alleles would help resolve species-dependent phenotypes. Finally, harmonization of histological, imaging, biochemical and proteomic endpoints across trials would enable meaningful comparisons among HSD17B13-directed agents and other emerging MASH therapies.

## 8 Conclusion

MASH is now understood as a disease of disrupted organelle communication rather than lipid excess alone. The LD-mitochondria interface governs the fate of fatty acids, directing them toward storage, oxidation or lipotoxic signalling. HSD17B13 has emerged as a key node at this interface: human loss-of-function variants confer protection against progressive liver disease, and the protein is amenable to both RNAi and small-molecule targeting. However, its physiological substrate, the balance between catalytic and scaffolding functions, and the origins of species-specific phenotypes remain unknown. The clinical success will require not only target engagement, but also patient selection and rational combination with agents that address complementary metabolic and fibrotic pathways. More broadly, this line of investigation provides a critical test of whether restoring inter-organelle metabolic coordination can be translated into a viable therapeutic paradigm in chronic liver disease.
